# Diterpenoids from the Roots of *Salvia yunnanensis*

**DOI:** 10.1007/s13659-015-0080-4

**Published:** 2015-12-14

**Authors:** Fan Xia, Chun-Yan Wu, Xing-Wei Yang, Xian Li, Gang Xu

**Affiliations:** School of Pharmaceutical Science and Yunnan Key Laboratory of Pharmacology of Natural Products, Kunming Medical University, Kunming, 650500 People’s Republic of China; State Key Laboratory of Phytochemistry and Plant Resources in West China, Kunming Institute of Botany, Chinese Academy of Sciences, Kunming, 650201 People’s Republic of China

**Keywords:** *Salvia yunnanensis*, Diterpenoid, Cytotoxicity

## Abstract

**Abstract:**

Two new diterpenoids, salyunnanins I and J (**1** and **2**), together with ten analogues, were isolated from the roots of *Salvia yunnanensis.* The structures of the new isolates, possessing different *neo*-clerodane and *seco*-abietane diterpenoid skeletons respectively, were elucidated on the basis of comprehensive spectroscopic data. All of the compounds were tested for the inhibitory activities against six human tumor lines in vitro, and several ones showed moderate cytotoxic activities.

**Graphical Abstract:**

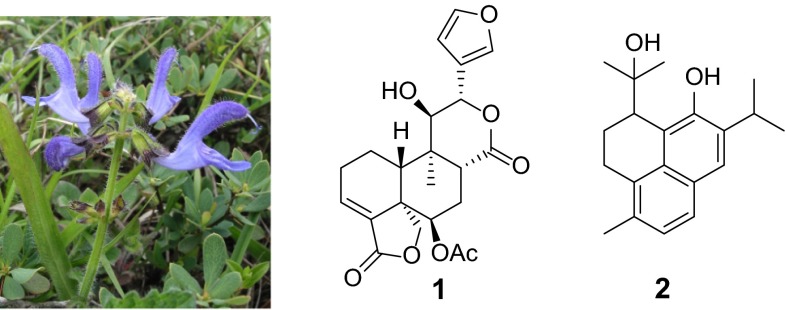

**Electronic supplementary material:**

The online version of this article (doi:10.1007/s13659-015-0080-4) contains supplementary material, which is available to authorized users.

## Introduction

**Fig. 1:**
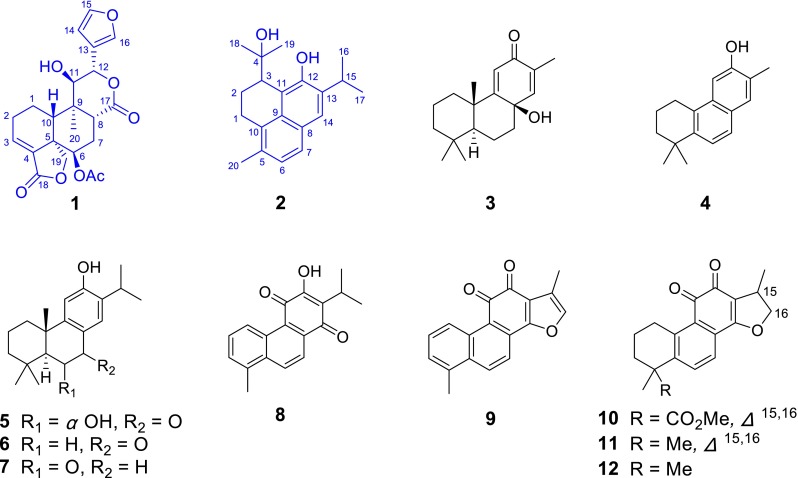
Structures of **1**–**12** isolated from *Salvia yunnanensis*

The genus of *Salvia* is a large pool of diterpenoids with structural diversity and biological properties [1–3]. Many diterpenoids with interesting bioactivities, such as tanshinone IIA, salvicine, neotanshinlactone, and salvinorin A, have been reported from this genus [4–6]. *Salvia yunnanensis* is a traditional Chinese herb used as the surrogate of *S. miltiorrhiza* (Danshen) for the treatment of various cardiovascular diseases [7]. Many bioactive abietane diterpenoids, especially a series of abietane type diterpene alkaloids, have been reported from this plant [8–10]. In a continuation of our research work on the diterpenoids from *Salvia* species, we examined the constituents of *S. yunnanensis* collected in Juhuacun traditional medicine market in the Yunnan province. As a result, two new diterpenoids, salyunnanins I and J (**1** and **2**), together with ten known analogues (**3**–**12**), were isolated (Fig. 1). It’s noteworthy that the structures of the new isolates were elucidated to possess *neo*-clerodane and *seco*-abietane diterpenoid skeletons respectively. All of the compounds were tested for the inhibitory activities against six human tumor lines in vitro, and several ones showed moderate cytotoxic activities. Herein, we report the isolation, structural elucidation, and the biological evaluation of all the obtained isolates.

## Results and Discussion

The acetone extract of the roots of *S. yunnanensis* was subjected to silica gel column chromatography eluting with petroleum ether-EtOAc to obtain seven fractions (I-VII), which were further purified by silica gel, Sephadex LH-20, and HPLC to afford two new diterpenoids (salyunnanins I and J, **1** and **2**) and ten known analogues: normiltioane (**3**) [11]; 5,6,7,8-tetrahydro-2,8,8-trimethyl-3-phenanthrenol (**4**) [12]; 6*α*-hydroxysugiol (**5**) [13]; sugiol (**6**) [14] 6-*oxo*-ferruginol (**7**) [15]; danshenxinkun B (**8**) [16]; tanshinone I (**9**) [17]; methyltanshinonate (**10**) [18]; tanshinone IIA (**11**) [19] and crypotanshinone (**12**) [20].

**Fig. 2:**
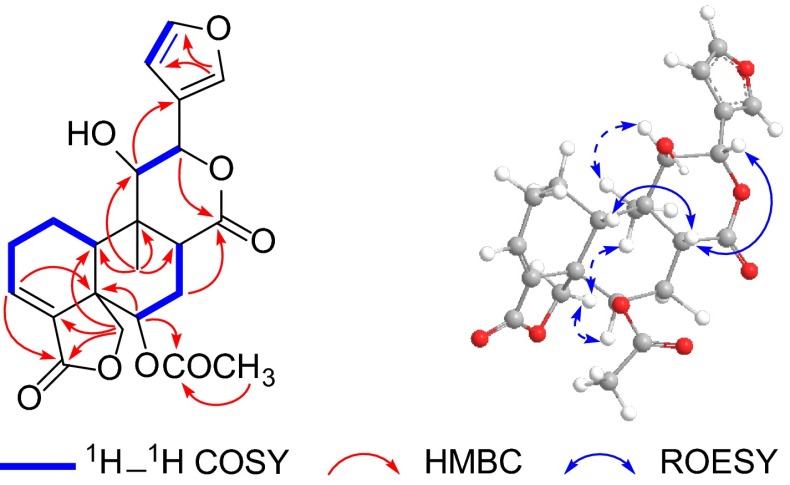
Key HMBC, ^1^H–^1^H COSY, and NOESY correlations of **1**

Salyunnanin I (**1**) was obtained as white powder. Its molecular formula C_22_H_24_O_8_ was established by its ^13^C NMR and HR-EIMS data ([M]^+^*m/z* 416.1467, calcd 416.1471). The IR spectrum exhibited absorption bonds due to hydroxyl (3425 cm^−1^) and lactone groups (1776 and 1747 cm^−1^). The ^13^C NMR and DEPT spectroscopic data (Table 1) exhibited 22 carbon signals assignable to two methyls (*δ*_C_ 15.4 and 21.0), four methylenes (including an oxygenated one at *δ*_C_ 71.5), nine methines (including four olefinic and three oxygenated ones at *δ*_C_ 69.6, 75.8 and 78.4), and seven quaternary carbons (including two olefinic ones and three lactonic carbonyls at *δ*_C_ 167.9, 170.0, and 173.5). Careful analysis of these ^13^C NMR data indicated that the characteristic signals for a *neo*-clerodane diterpenoid of two quaternary carbons (*δ*_C_ 49.9, C-5; 40.7, C-9), a methine (*δ*_C_ 39.6, C-10), an oxygenated methylene (*δ*_C_ 71.5, C-19), and a substituted furan ring (*δ*_C_ 125.5, C-13; 109.8, C-14; 144.8, C-15; 141.1, C-16) were all presented. These evidence, conjugated with some same type of diterpenoid were previously isolated in our laboratory [21], suggested that compound **1** could be a *neo*-clerodane diterpenoid. The ^1^H and ^13^C NMR data (Table 1) of **1** were very similar to those of dugesin E [21], except that the methylene carbon at *δ*_C_ 43.4 (C-11) in dugesin E was absent, while a down-field methine at *δ*_C_ 75.8 was instead present in **1**. The difference implied that compound **1** was a C-11 oxygenated product of dugesin E. This assumption was supported by the HR-EIMS data, and further supported by the correlation from *δ*_H_ 0.80 (Me-20) to *δ*_C_ 75.8 (C-11) in the HMBC spectrum. Other partial planar structure of **1** was identical to those of dugesin E by detailed analysis of ^1^H–^1^H COSY and HMBC spectra (Fig. 2).

**Table 1:** ^13^C (125 MHz) NMR spectral data (*δ* in ppm and *J* in Hz) of **1** and **2**

No.	**1** ^a^		**2** ^b^	
	*δ*_C_ type	*δ*_H_, mult.	*δ*_C_ type	*δ*_H_, mult.
1	20.9 CH_2_	1.91, brd (12.8)	23.4 CH_2_	2.89, dd (16.9, 5.6)
		1.28, m		2.77, m
2	27.6 CH_2_	2.32, m	24.3 CH_2_	2.33, m
		2.23, m		1.92, m
3	139.9 CH	6.79, dd (7.9, 2.0)	42.6 CH	3.50, brd (5.8)
4	136.0 C		78.9 C	
5	49.9 C		131.2 C	
6	69.6 CH	5.24, brd (5.3)	125.6 CH	7.09, d (8.3)
7	24.8 CH_2_	2.28, m	125.4 CH	7.51, d (8.3)
		2.10, m		
8	39.0 CH	3.20, dd (12.4, 4.9)	127.6 C	
9	40.7 C		129.7 C	
10	39.6 CH	2.90, m	129.9 C	
11	75.8 CH	3.90, t (6.2)	117.3 C	
12	78.4 CH	5.19, d (6.2)	150.6 C	
13	125.5 C		137.6 C	
14	109.8 CH	6.58, brs	123.5 CH	7.49, s
15	144.8 CH	7.69, brs	27.7 CH	3.45, sept (6.8)
16	141.1 CH	7.60, brs	23.1 CH_3_	1.27, d (6.8)
17	173.5 C		22.5 CH_3_	1.35, d (6.8)
18	167.9 C		29.6 CH_3_	1.56, s
19	71.5 CH_2_	4.44, d (8.9)	28.4 CH_3_	0.93, s
		4.18, d (8.9)		
20	15.4 CH_3_	0.80, s	19.7 C	2.34, s
COMe	170.0 C			
COMe	21.0 CH_3_	1.99, s		

^a^Recorded in acetone-*d*_6_

^b^Recorded in CDCl_3_

In the NOESY spectrum, the NOE correlations of Me-20 with H_2_-19, of H-19a (*δ*_H_ 4.44) with H-6, of H-10 with H-8, and of H-8 with H-12 indicated that the relative configurations of C-5, C-6, C-8, C-9, C-10, and C-12 of **1** were the same as dugesin E. In addition, the strong NOE contact between Me-20 and H-11 defined the *α*-orientation of H-11 (Fig. 2). Hence, the structure of **1** was elucidated and named salyunnanin I.

**Fig. 3:**
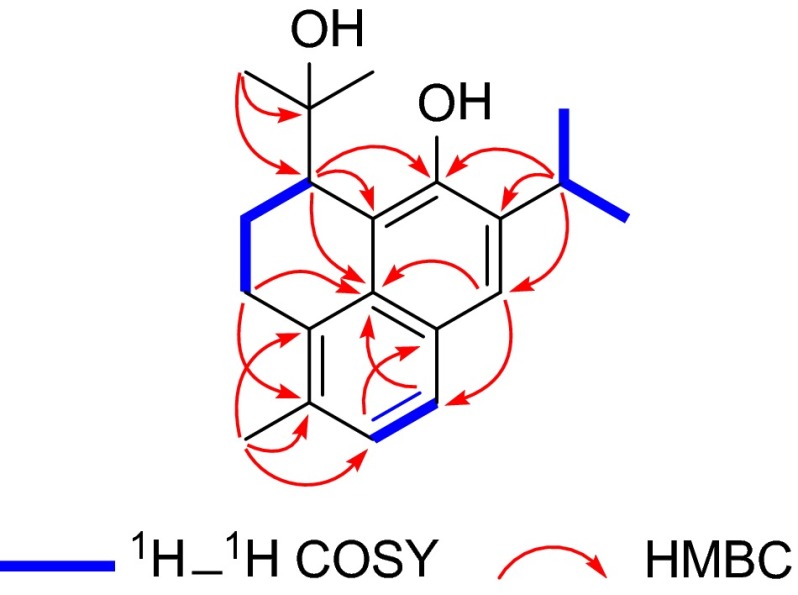
Key HMBC and ^1^H–^1^H COSY correlations of **2**

Salyunnanin J (**2**) was assigned the molecular formula C_20_H_26_O_2_ by its ^13^C NMR and HR-ESIMS (*m/z* 321.1835, [M + Na]^+^) data, 18 mass units more than that of (3-rac)-4,12-Epoxy-3,11-cyclo-4,5-seco-20(10 → 5)-*abeo*-abieta-5(10),6,8,11,13-pentaene, a cyclization product of aethiopionone [22]. Comparing of the NMR spectroscopic data of **2** (Table 1) with those of the cyclization product, about 14.7 and 1.7 ppm up-field shifts for C-4 (*δ*_C_ 78.9) and C-12 (*δ*_C_ 150.6), respectively, were found in **2**, which indicated that **2** was a hydrolytic derivative of the known compound. The HR-ESIMS data (*m/z* 321.1835 [M + Na]^+^, calcd for 321.1830) supported this assumption. Furthermore, the correlations of H-1 (*δ*_H_ 2.89, 2.77)/H-2 (*δ*_H_ 2.33, 1.92)/H-3 (*δ*_H_ 3.50) and H-6 (*δ*_H_ 7.09)/H-7 (*δ*_H_ 7.51) in the ^1^H–^1^H COSY spectrum, together with the correlations from H-1 to C-5 (*δ*_C_ 131.2) and C-9 (*δ*_C_ 129.7), from H-3 to C-9, C-11 (*δ*_C_ 117.3), and C-12 (*δ*_C_ 150.6), from H-15 (*δ*_H_ 3.45) to C-12, C-13 (*δ*_C_ 137.6), and C-14 (*δ*_C_ 123.5), and from Me-20 (*δ*_H_ 2.34) to C-5, C-6 (*δ*_C_ 125.6), and C-10 (*δ*_C_ 129.9) in the HMBC spectrum further confirmed the structure of **2** (Fig. 3).

*Salvia* (including 700–1050 species) is the biggest genus in the medicinal important Labiatae family and widely distributed in the world [23, 24]. These plants are also a rich source of various diterpenoids, especially the clerodane and abietane diterpenoids with diverse skeletons. Structurally, the clerodane diterpeoids are typical bicyclic diterpenoids, while abietane are tricyclic diterpenoids. Accordingly, their biogenetic pathways are totally different with each other. Detailed investigation of the literatures revealed an interesting relationship between the diterpenoids constituents and its taxonomy of *Salvia* plants: the clerodane diterpenoids are usually reported from plants distributed in America, while abietane and related diterpenoids are commonly reported from plants in Asia. Up to now, these two types of diterpenoids have only been reported from a few *Salvia* plants, such as *S. cardiophylia* and *S. lavanduloides*, simultaneously [25, 26].

All of the compounds were tested for their cytotoxic effects against six human cancer cell lines (Hela, NCI-H460, PC3, KB-3-1, MCF-7, and K562) using a previously described MTT method [27]. As shown in Table 2, compounds **5**, **6**, and **11** exhibited moderate activities.

**Table 2:** Cytotoxcities of the isolates on six cancer cell lines with IC_50_ values (μM)

Compound^a^	HeLa	KB-3-1	NCI-H 460	PC3	MCF-7	K562
**5**	7.7	7.7	7.4	6.0	8.7	5.0
**6**	10.9	9.1	8.9	10.5	11.4	6.7
**7**	>20	>20	17.2	>20	>20	15.9
**9**	16.7	12.8	>20	5.2	>20	6.1
**11**	10.1	9.2	>20	8.9	>20	5.5
**12**	17.1	10.3	>20	>20	>20	13.6
Paclitaxel^b^	0.01	0.003	0.004	0.007	0.21	0.005

^a^Other selected ones not listed in the table were inactive (IC_50_ > 20 μM) for all cell lines

^b^Paclitaxel was used as positive controls

In conclusion, totally twelve diterpenoids, including two new ones (salyunnanins I and J), were characterized from *S. yunnanensis* in this study. Eleven of the isolates were abietane diterpenoids, while salyunnanin I (**1**) was elucidated to be a *neo*-clerodane diterpenoid. In the bioassay of these isolates, several compounds exhibited moderate cytotoxic activities.

## Experimental

### General Experimental Procedures

Optical rotations were measured on a Jasco P-1020 polarimeter. UV spectra were detected on a Shimadzu UV-2401PC spectrometer. IR spectra were determined on a Bruker FT-IR Tensor-27 infrared spectrophotometer with KBr disks. 1D and 2D NMR spectra were recorded on DRX-500 spectrometers using TMS as an internal standard. Unless otherwise specified, chemical shifts (δ) were expressed in ppm with reference to the solvent signals. ESIMS and HR-EIMS analysis were carried out on Waters Xevo TQS and Waters AutoSpec Premier P776 mass spectrometers, respectively. Semi-preparative HPLC was performed on an Agile 1100 HPLC with a Zorbax SB-C_18_ (9.4 × 250 mm) column. Silica gel (100–200 and 200–300 mesh, Qingdao Marine Chemical Co., Ltd., PR China), and Amphichroic RP-18 gel (40–63 μm, Merck, Darmstadt, Germany) and MCI gel (75–150 μm, Mitsubishi Chemical Corporation, Tokyo, Japan) were used for column chromatography.

### Plant Material

The roots of *S. yunnanensis* were collected in Kunming, Yunnan Province, PR China, in October 2010. The plant was identified by Dr. En-De Liu, Kunming Institute of Botany, Kunming, PR China. A voucher specimen was deposited at the Kunming Institute of Botany with identification number 201010S01.

### Extraction and Isolation

The air-dried powdered material (20 kg) was extracted with acetone (3 × 50 L × 24 h) at room temperature and the solution was evaporated in vacuum to give a crude extract (1.2 kg). The extract was subjected to column chromatography over silica gel, eluting with CHCl_3_–EtOAc to afford seven fractions (I–VII). Fraction II (311 g) was further chromatographed on silica gel (eluting with a gradient of EtOAc in petroleum ether) to yield seven subfractions (A–G). Subfraction B (54 g) was separated over an MCI-gel column (MeOH–H_2_O from 3:2 to 10:0) to obtain five fractions (Fr. B1**–**B5). Fr. B1 (12.5 g) was then chromatographed on a silica gel column eluted with petroleum ether–Chloroform (from 10:1 to 0:10), to yield four fractions (Fr. B1a**–**B1d). Fr. B1a (3.3 g) was repeatedly subjected to silica gel columns eluted with petroleum ether–EtOAc (from 500:1 to 2:1), and was then further purified by semi-preparative HPLC (MeOH–H_2_O, 85:15) to afford compounds **1** (6 mg) and **8** (4 mg). Fr. B2 (22 g) was isolated over an MCI gel column (MeOH–H_2_O from 75:25 to 100:0) to provide **2** (15 mg) and **3** (3 mg). Similarly, compounds **4** (35 mg), **5** (10 mg), **6** (18 mg), and **7** (10 mg) from subfraction C (32 g), and **9** (5 mg), **10** (430 mg), **11** (12 g), **12** (5 g) from subfraction D (120 g) were obtained.

### Cytotoxicity Assays

The following human tumor cell lines were used: HeLa, NCI-H460, PC3, KB-3-1, MCF-7, and K562, which were kindly provided by Prof. David WF Fong of Hong Kong Baptist University. All cells were cultured in RPMI-1640 or DMEM medium [Gibco, Invitrogen (Shanghai), China] supplemented with 10 % fetal bovine serum [Gibco, Invitrogen (NY), USA] at 37 °C in a humidified atmosphere with 5 % CO_2_. Cell viability was assessed by conducting colorimetric measurements of the amount of insoluble formazan formed in living cells based on the reduction of 3-(4,5-dimethylthiazol-2-yl)-2,5-diphenyltetrazolium bromide (MTT) (Sigma, St. Louis, MO, USA) [28]. Briefly, 100 μL of adherent cells (HeLa, NCI-H460, PC3, KB-3-1, MCF-7) was seeded into each well of a 96-well cell culture plate and allowed to adhere for 24 h before test compound addition, while suspended cells (K562) were seeded just before test compound addition, both with an initial density of 1 × 10^5^ cells/mL in 100 μL of medium. Each tumor cell line was exposed to the test compound at various concentrations in triplicate for 48 h, with paclitaxel (Sigma) as positive control. After the incubation, 10 μL of MTT (5 mg/mL in PBS) was added to each well, and the incubation continued for 4 h at 37 °C. After removing the medium, 100 μL per well of DMSO was added to dissolve the residue and the optical density was measured at 492 nm in a 96-well microtiter plate reader (Bio-Rad 680). The IC_50_ value of each compound was calculated by Reed and Muench’s method [27].

#### Salyunnanin J (**1**)

White powder; [α]_D_^21^ −95.7 (*c* 0.22, MeOH); UV (MeOH) *λ*_max_ (log *ε*): 208 (4.10), 249 (3.24) nm; IR (KBr) *ν*_max_ 3425, 1776, 1747, 1374, 1241, 1189, 1169, 1024, 1003 cm^−1^; ^1^H and ^13^C NMR data, see Table 1; negative ESIMS *m/z* 451 [M + Cl]^−^; HR-EIMS *m/z* 416.1467 [M]^+^ (calcd for C_22_H_24_O_8_, 416.1471).

#### Salyunnanin K (**2**)

Yellow powder; [α]_D_^16^ −12.6 (*c* 0.10, MeOH); UV (MeOH) *λ*_max_ (log *ε*): 195 (4.22), 237 (4.69), 288 (3.68), 332 (3.36) nm; IR (KBr) *ν*_max_ 3431, 2963, 2923, 2855, 1626, 1461, 1417, 1249, 1231 cm^−1^; ^1^H and ^13^C NMR data, see Table 1; EIMS *m/z* 298 [M]^+^; HR-ESIMS *m/z* 321.1835 [M + Na]^+^ (calcd for C_20_H_26_O_2_Na, 321.1830).

## Electronic supplementary material

Supplementary material 1 (PDF 711 kb)
