# Supplementary material for: Diterpenoids from the Roots of Salvia yunnanensis
Source: Nat Prod Bioprospect. 2015 Dec 14;5(6):307–12. doi: 10.1007/s13659-015-0080-4 (PMC4681706; doi:10.1007/s13659-015-0080-4)
Supplement: Supplementary file 1 — Supplementary material 1 (PDF 711 kb) [file 13659_2015_80_MOESM1_ESM.pdf]

Diterpenoids from the Roots of *Salvia yunnanensis*

Fan Xia, Chun-Yan Wu, Xing-Wei Yang, Xian Li, and Gang Xu

Supplementary material

Table of Contents

- Figures S1–S15: The original NMR and MS spectra of the new compounds.

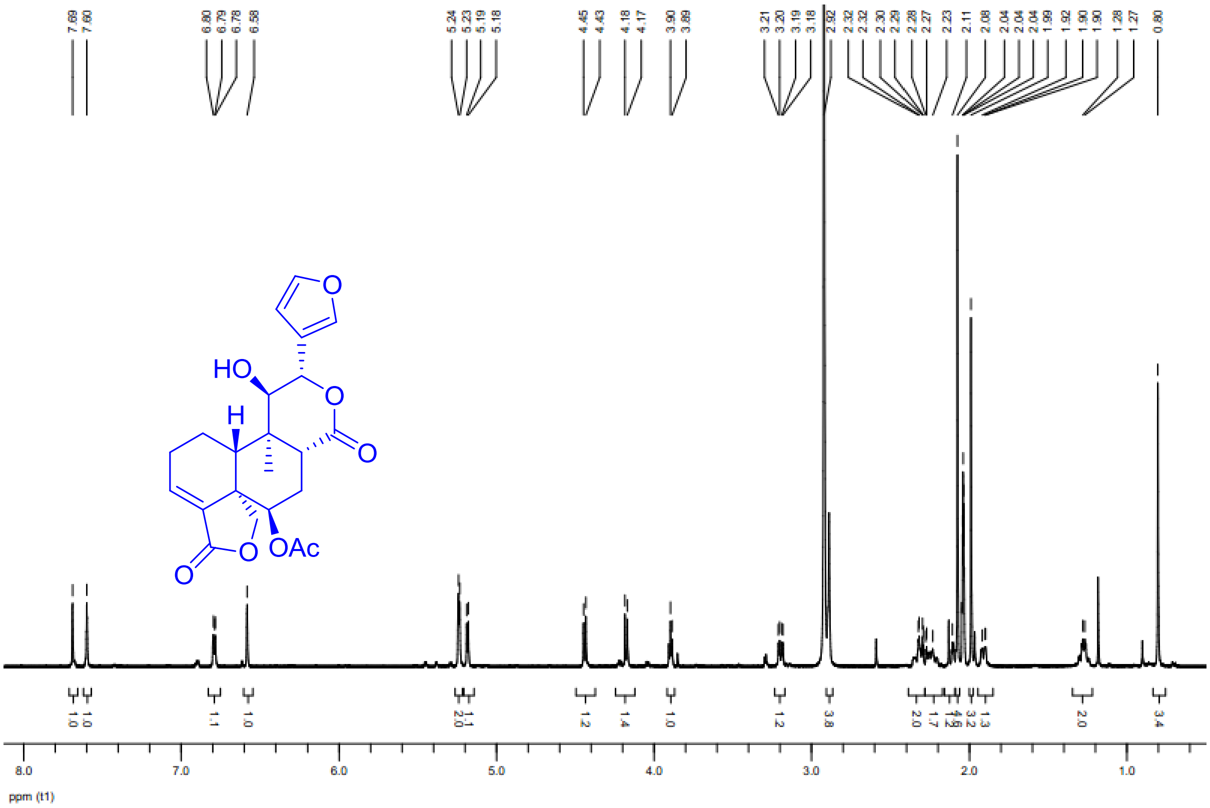

Figure S1. ^{1}H (in acetone-d_6) spectrum of salyunnanin I (**1**).

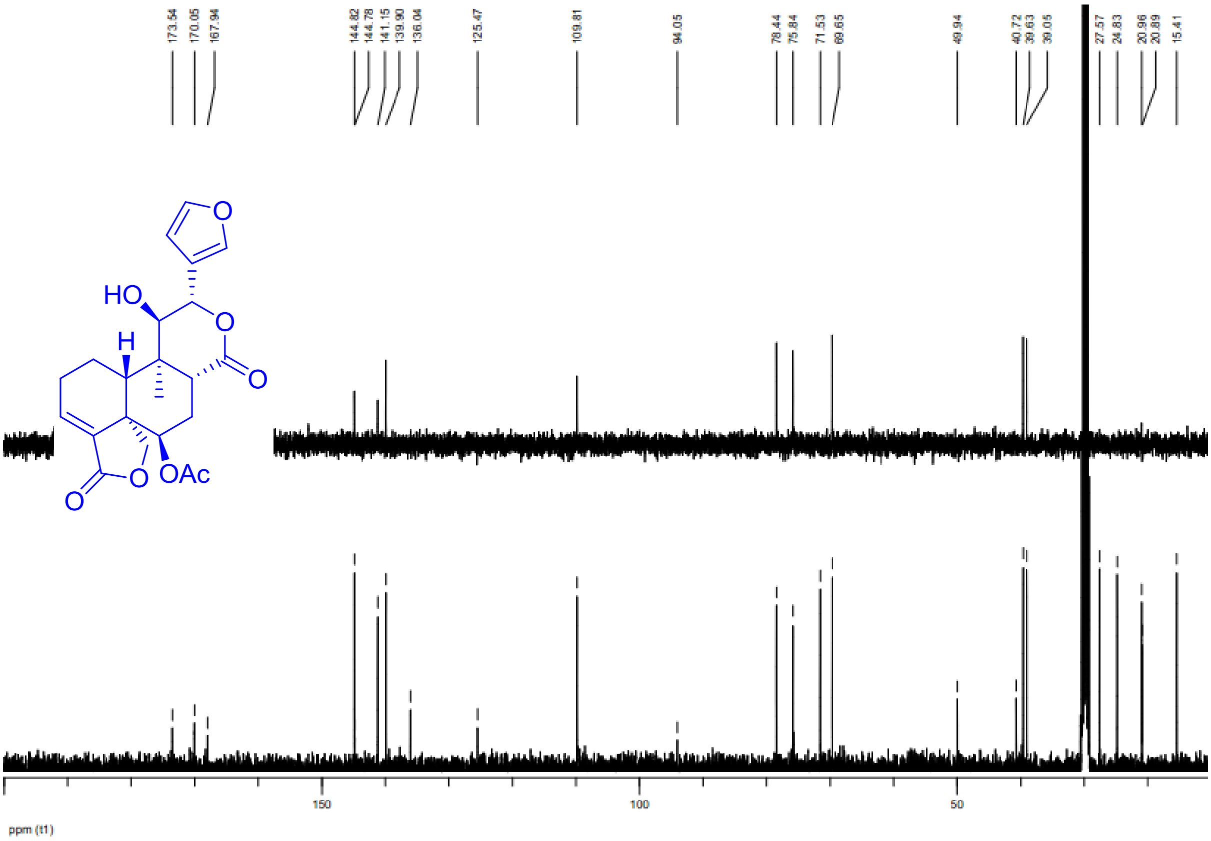

Figure S2. ^{13}C and DEPT (in acetone-d_6) spectra of salyunnanin I (**1**).

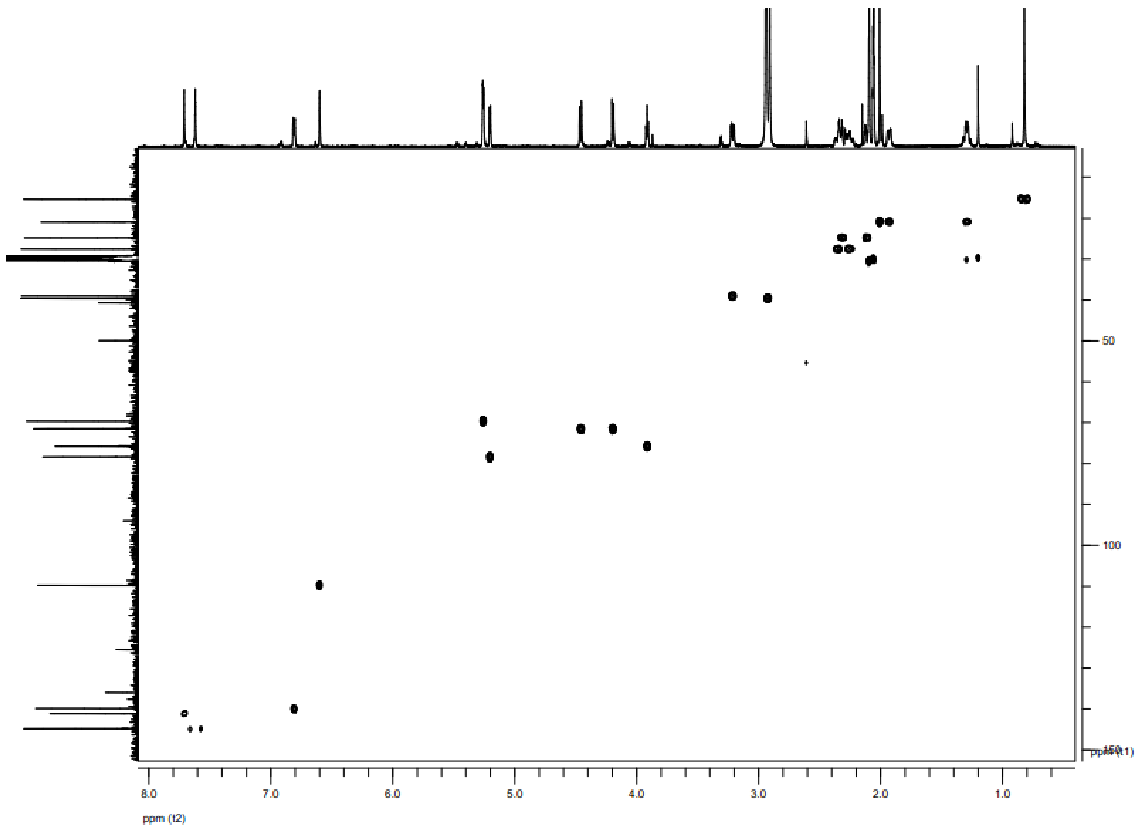

Figure S3. HSQC spectrum of salyunnanin I (**1**).

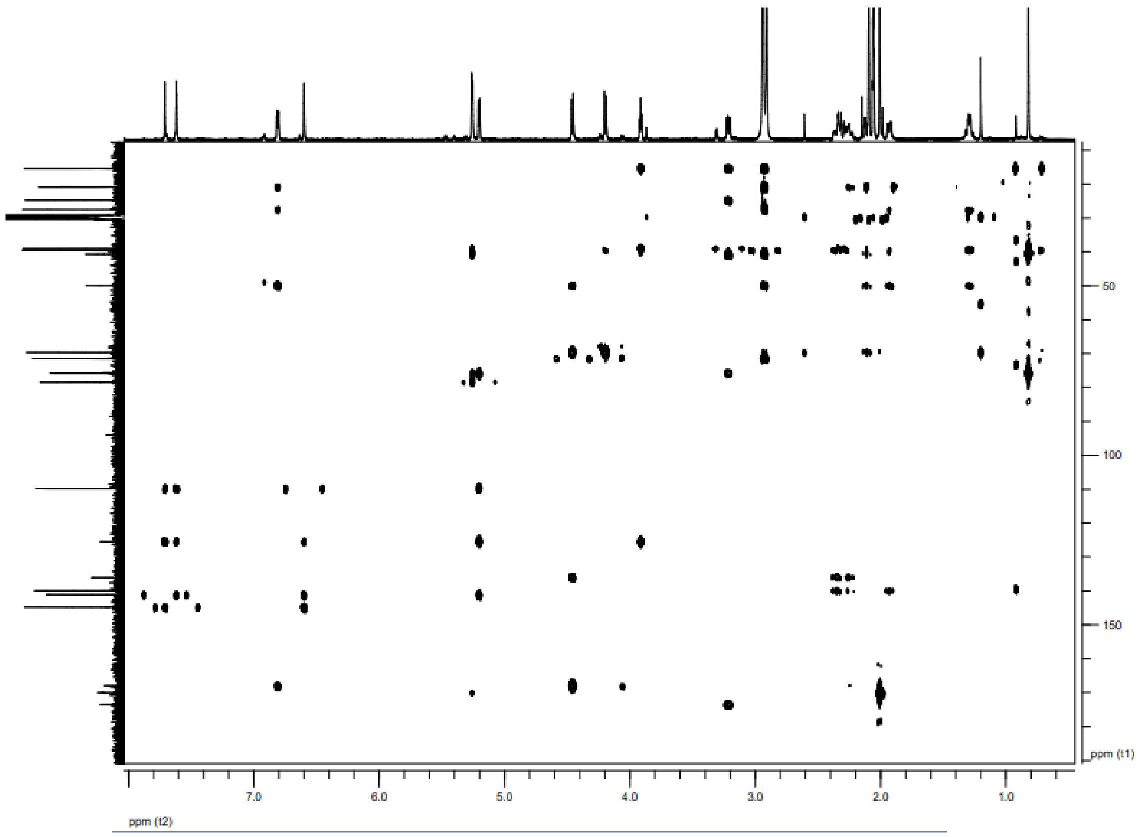

Figure S4. HMBC spectrum of salyunnanin I (**1**).

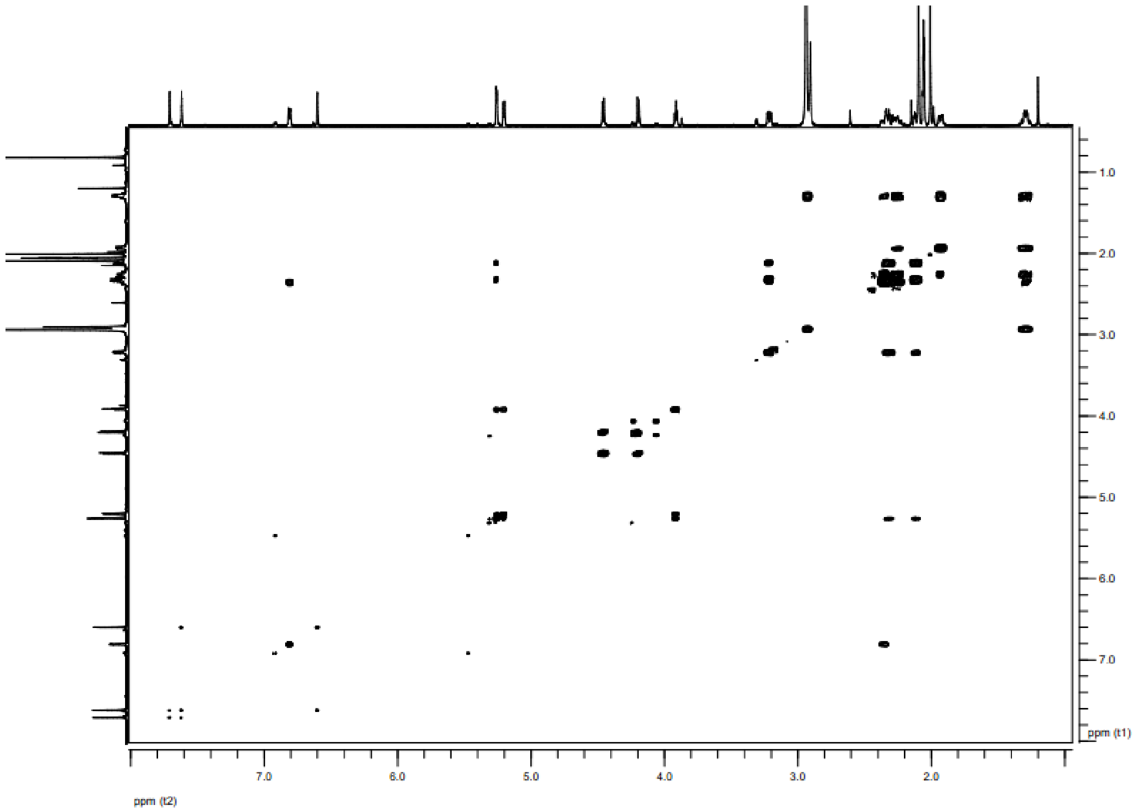

Figure S5. $^{1}H–^{1}H$ COSY spectrum of salyunnanin I (**1**).

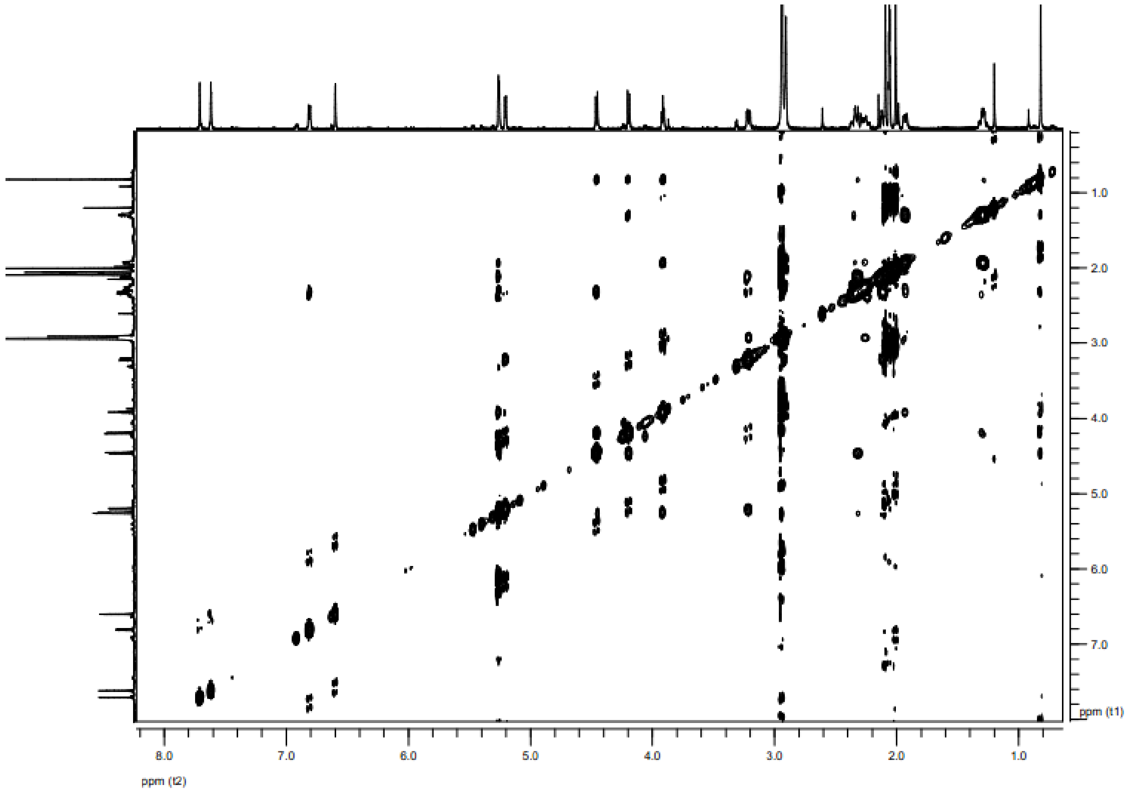

Figure S6. NOESY spectrum of salyunnanin I (**1**).

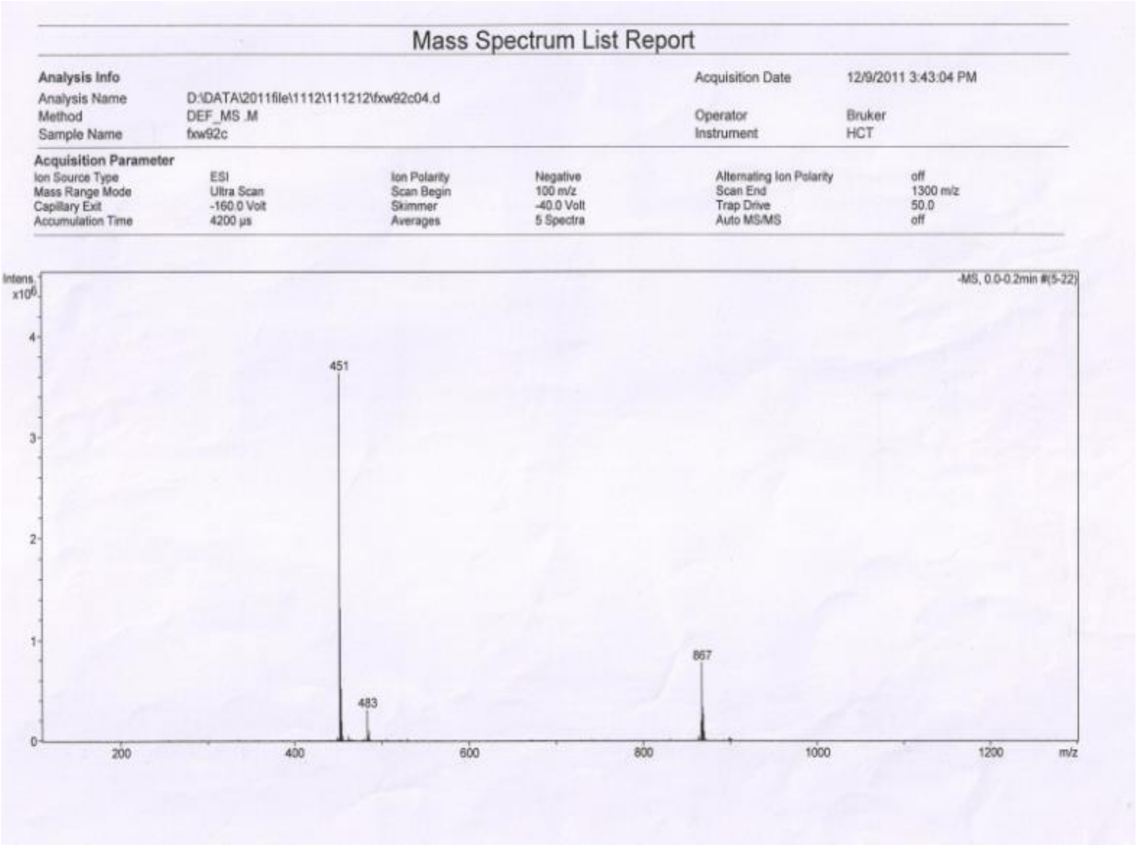

Figure S7. ESIMS spectrum of salyunnanin I (**1**).

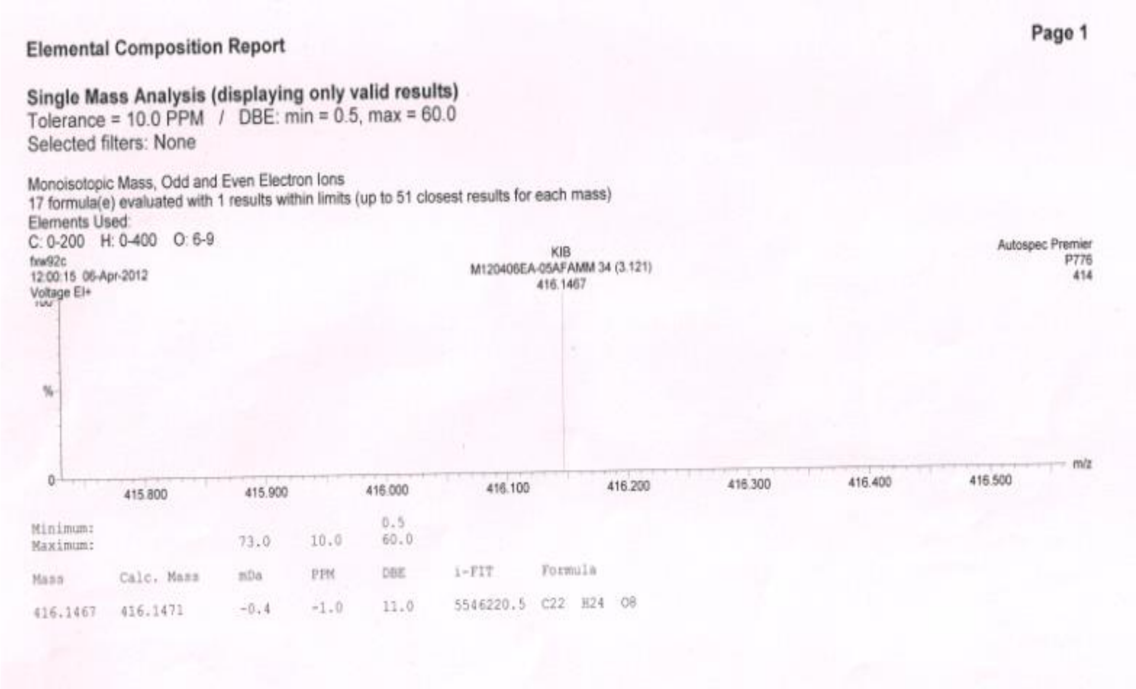

Figure S8. HR-EIMS spectrum of salyunnanin I (**1**).

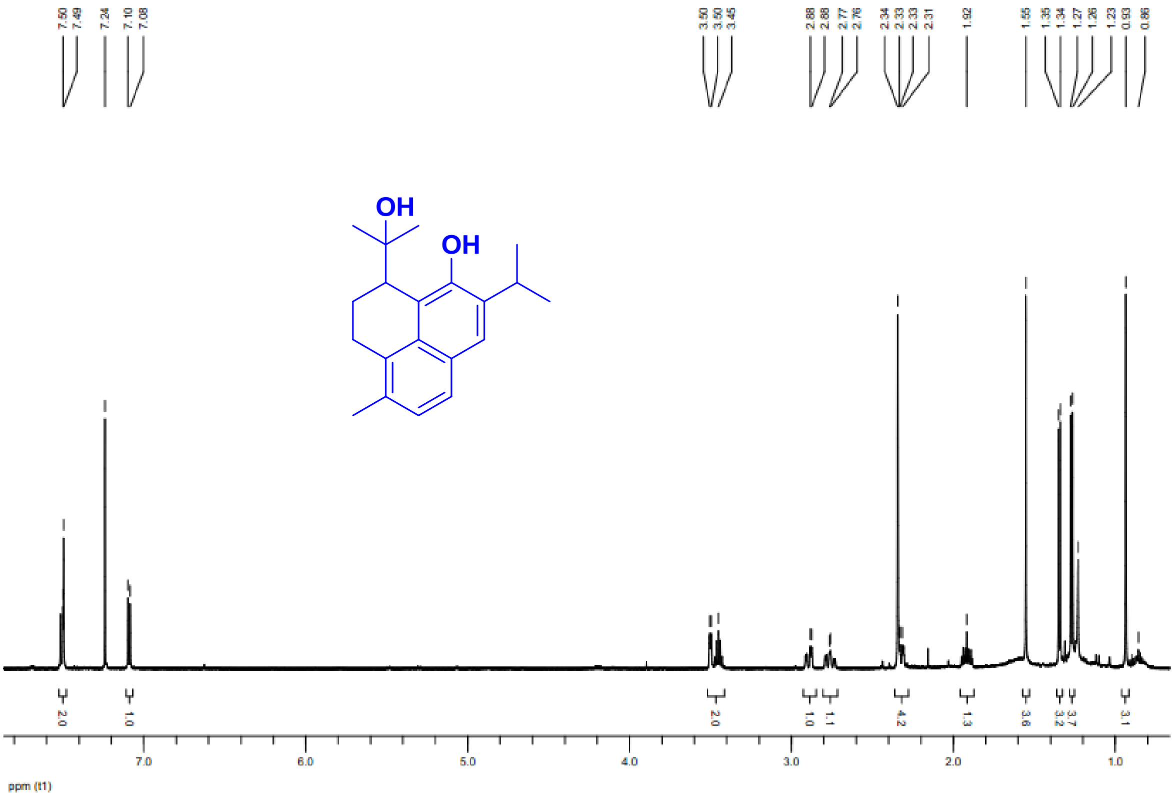

Figure S9. ^{1}H (in $CDCl_3$) spectrum of salyunnanin J (**2**).

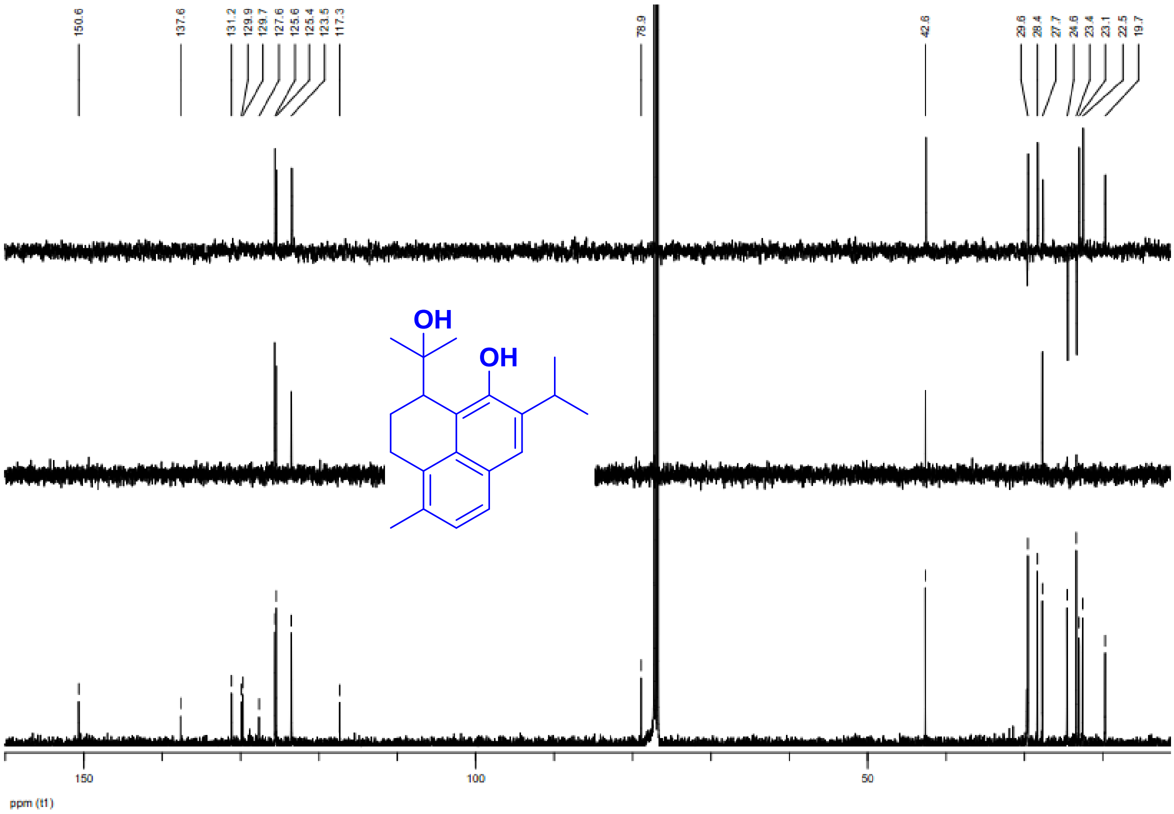

Figure S10. ^{13}C and DEPT (in $CDCl_3$) spectra of salyunnanin J (**2**).

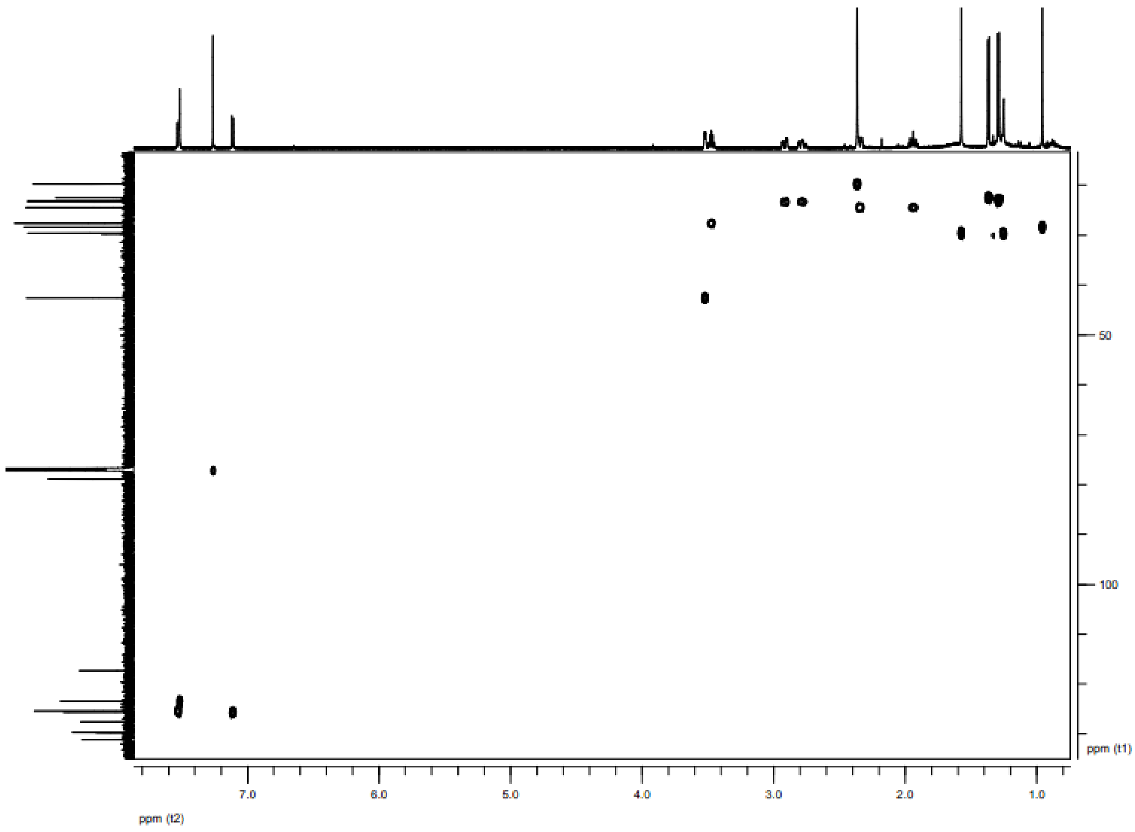

Figure S11. HSQC spectrum of salyunnanin J (**2**).

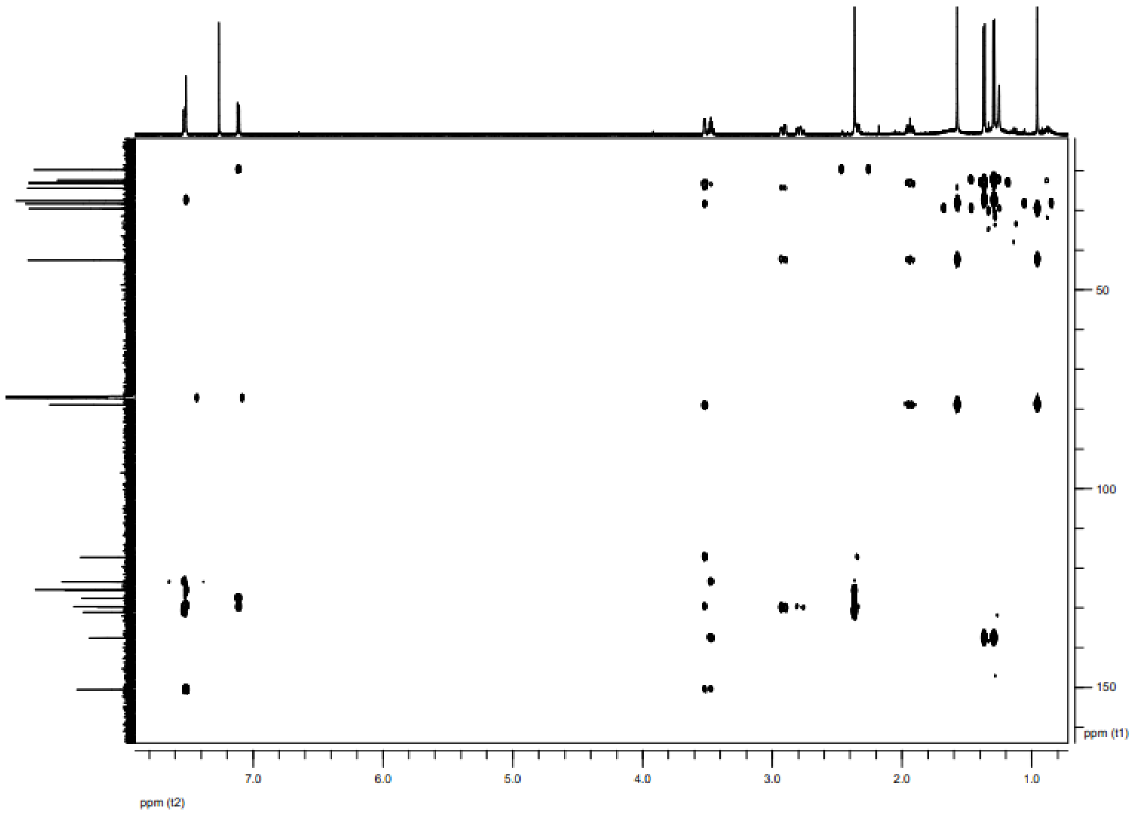

Figure S12. HMBC spectrum of salyunnanin J (**2**).

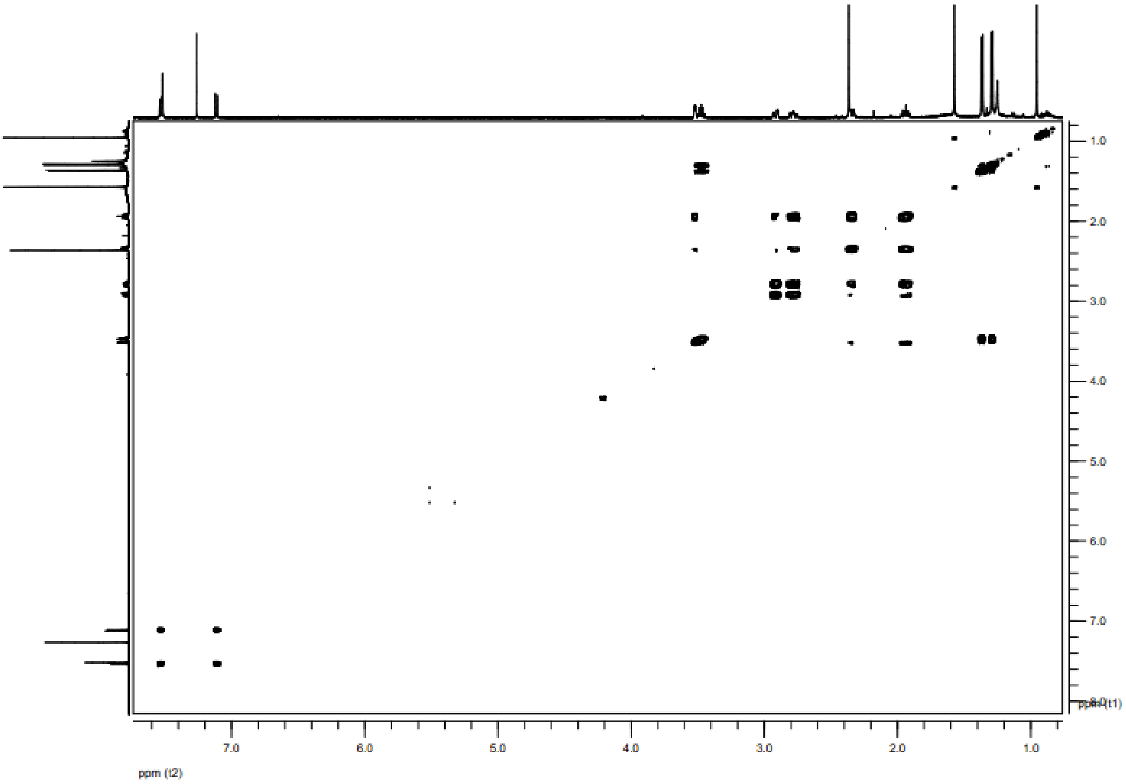

Figure S13. ^{1}H–^{1}H COSY spectrum of salyunnanin J (**2**).

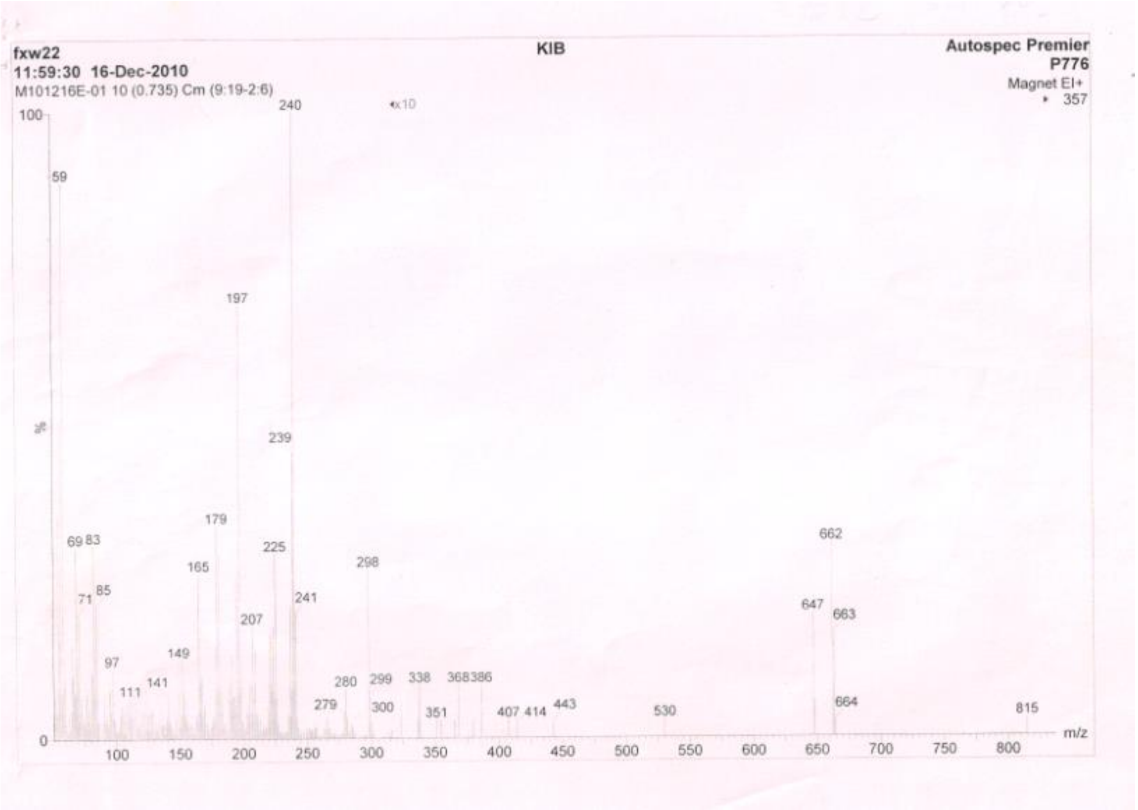

Figure S14. EIMS spectrum of salyunnanin J (**2**).

Acq. Date: Wednesday, February 08, 2012 Acq. Time: 13:31

Sample Name: 120207ESIA fxw22

Elemental composition calculator

Target m/z: +321.1835 amu
Tolerance: +10.0000 ppm
Result type: Elemental
Max num of results: 1000
Min DBE: -10.0000 Max DBE: +60.0000
Electron state: OddAndEven
Num of charges: 0
Add water: N/A
Add proton: N/A
File Name: 120207ESIA fxw22.wiff

	Elements	Min Number	Max Number
1	2H	0	0
2	Br	0	0
3	C	0	200
4	Cl	0	0
5	F	0	0
6	H	0	400
7	I	0	0
8	K	0	0
9	N	0	0
10	Na	1	1

Acq. Date: Wednesday, February 08, 2012 Acq. Time: 13:31

Sample Name: 120207ESIA fxw22

	Elements	Min Number	Max Number
11	O	0	4
12	P	0	0
13	Pt	0	0
14	S	0	0
15	Si	0	0

	Formula	Calculated m/z (amu)	mDa Error	PPM Error	DBE
1	C20 H26 O2 Na	321.1830	0.4499	1.4010	7.5

Figure S15. HR-ESIMS spectrum of salyunnanin J (**2**).
